# Neuropsychological outcome in survivors of congenital diaphragmatic hernia at 5 years of age, what does it tell?

**DOI:** 10.1007/s00431-022-04696-1

**Published:** 2022-12-24

**Authors:** Sophie de Munck, Suzan C. M. Cochius-den Otter, J. Marco Schnater, Joost van Rosmalen, Nina C. J. Peters, Annabel P. J. M. van Gils-Frijters, Neeltje E. M. van Haren, Saskia J. Gischler, Hanneke IJsselstijn, André B. Rietman

**Affiliations:** 1 Department of Pediatric Surgery and Intensive Care, Erasmus MC-Sophia Children’s Hospital, Rotterdam, The Netherlands; 2 Department of Biostatistics, Erasmus MC, Rotterdam, The Netherlands; 3 Department of Epidemiology, Erasmus MC, Rotterdam, The Netherlands; 4 Department of Obstetrics and Gynaecology, Division of Obstetrics and Foetal Medicine, Erasmus MC, Rotterdam, The Netherlands; 5 Department of Child and Adolescent Psychiatry and Psychology, Erasmus MC-Sophia Childrens Hospital, Rotterdam, The Netherlands

**Keywords:** Congenital diaphragmatic hernia, Pediatric surgery, Pediatric intensive care, Neuropsychological outcome, Long-term follow-up

## Abstract

Previous studies have frequently reported neurocognitive deficits in children born with congenital diaphragmatic hernia (CDH) at school age, which may contribute to academic difficulties. Yet, age at onset of these deficits is currently unknown. We evaluated neurocognitive skills with possible determinants in preschool children born with CDH. Eligible 5-year-old children born with CDH (2010–2015) who participated in our prospective structural follow-up program were included. We used the WPPSI-III to assess intelligence, subtests of the Kaufman-ABC for memory, and NEPSY-II to assess inhibition and attention. We included 63 children. Their test scores generally were within or significantly above normal range: total IQ = 103.4 (15.7) (*p* = 0.13); Verbal memory = 10.2 (2.8) (*p* = 0.61); Visuospatial memory = 11.4 (2.6) (*p* < 0.01); Inhibition = 10.5 (2.2), (*p* = 0.10). In univariable analyses, length of ICU-stay was negatively associated with IQ, and maximum vasoactive inotropic score and open repair were negatively associated with inhibition skills. In multivariable regression analysis, the latter association remained (*B* = 5.52, *p* = 0.04 (CI 0.32–10.72)).

*   Conclusions: *In these tested 5-year-old children born with CDH, neuropsychological outcome was normal on average. While problems in 8-year-olds are common, we did not detect onset of these problems at age 5. Yet, we cannot rule out that this cohort had a relatively mild level of disease severity; therefore, conclusions should be interpreted with caution. However, given the growing-into-deficit hypothesis, meaning that deviant brain development in early life is revealed once higher cognitive brain functions are demanded, follow-up should be conducted up to school age, and preferably beyond.

**Table:** 

**What is Known:** *• Children born with CDH are at risk for academic difficulties at school age.* *• Whether these difficulties can be detected already before school age is unknown.*	
**What is New:** *• At age 5 years, intelligence, inhibition, attention, and memory skills were all within normal range, or even above, in children with CDH. This is supportive of the growing-into-deficit hypothesis in this patient population.* *• Those who underwent open surgical correction had poorer inhibition skills than those who were corrected with minimal access surgery.*

**Supplementary Information:**

The online version contains supplementary material available at 10.1007/s00431-022-04696-1.

## Introduction

With a prevalence of 2.6 per 10,000 births [1], congenital diaphragmatic hernia (CDH) is a rare condition, which is life-threatening and often requires a prolonged stay on the intensive care unit (ICU) [2, 3]. Fortunately, survival rates of children born with CDH are increasing, but so is the occurrence of developmental delays, which may stretch into adulthood and beyond [4, 5]. Delays concern both motor function and neurocognitive domains which impact daily life; particularly memory and attention deficits have been reported at school age, possibly contributing to academic difficulties [5–7]. Of note, those educational problems tend to become overt only around 8 years of age, possibly because of the stronger appeal on a diversity of brain functions that are needed for cognitive processing. The latter phenomenon has been coined the “growing-into-deficit” phenomenon, as deviant brain development in early life is revealed not until those higher cognitive brain functions are demanded [8]. While intelligence has been investigated in CDH-survivors at preschool age [9, 10], more specific neurocognitive functions such as memory have not been studied extensively. It is therefore currently unknown whether these deficits are already detectable at an earlier stage. If so, timely detection is important, because this would provide the opportunity to intervene and possibly prevent children from educational delay [11]. Moreover, clinical variables predictive of cognitive deficits are not yet available, while those can be of additional value in the search for possible early interventions. We hypothesized that memory and attention problems are present earlier in life and can be detected by using comprehensive neurocognitive assessments [12–14]. We aimed to investigate intelligence, memory, inhibition, and attention together with clinical predictors in preschool children born with CDH.

## Methods

### Inclusion criteria

We included data of CDH-survivors born between February 2010 and November 2015 who had joined the structural follow-up program at the Erasmus MC-Sophia Children’s Hospital. This multidisciplinary program includes assessments of neurocognitive function, motor function, lung function, and exercise capacity. At 30 months, and at 5, 8, 12, and 17 years of age, a psychologist evaluates neurodevelopment and cognitive functioning. In case of emerging problems, children are offered extra help [5, 15].

### Exclusion criteria

Children were excluded if they had been diagnosed with CDH later than 7 days post-partum, when the anomaly appeared to be a para-esophageal hernia or an eventration of the diaphragm, when diagnosed with a genetic syndrome known to affect neurodevelopment, or in case of severe neurologic or developmental impairment, due to which assessments could not be administered.

### Neuropsychological assessment

All cognitive tests were age-appropriate and offered to all participants in our follow-up program as part of routine care (Appendix). They were administered by certified and experienced psychologists. Norm-scores of all tests are based on a representative sample of children without disabilities. Intelligence was assessed with the Dutch version of the Wechsler Preschool and Primary Scale of Intelligence–III (WPPSI-III-NL) [16], which has an average population mean score for intelligent quotient (IQ) of 100, with a standard deviation (SD) of 15. The WPPSI-III-NL covers three domains of intelligence, i.e., performance IQ (3 subtests), verbal IQ (3 subtests), and processing speed (2 subtests). Total IQ is based on seven subtests. To assess both inhibition and attention, several subtests from the second version of A Developmental NEuroPSYchological Assessment (NEPSY-II-NL) were used [17], i.e., Inhibition-Naming (NEPSY-II-NL-IN), Inhibition-Inhibition (NEPSY-II-NL-II), and Auditory Attention (NEPSY-II-NL-AA). During Inhibition-Naming, children are given a sheet with target pictures and are asked to name the shape or direction of the target pictures as accurately and rapidly as possible. Performance on the task is reported as a norm score, with a mean score of 10 and an SD of 3, representing the time needed to finish the task. Low scores indicate slow processing speed or poor naming ability.

For Inhibition-Inhibition, children must rapidly and accurately name the opposite shape or direction of the target pictures. Completion time and the number of mistakes made are expressed in percentile categories (0–10 and > 10). Many mistakes indicate an impulsive approach, whereas a longer completion time suggests that requirements for inhibition slow down processing speed, indicating a problem with inhibition. The number of mistakes made in both Inhibition-Naming and Inhibition-Inhibition is added up, resulting in a percentile category representing the level of performance.

The Auditory Attention task was used to assess selective and sustained attention. In this task, children must selectively respond to auditory target words. The result is represented by the number of correct responses and is expressed in percentile categories. Lower scores indicate increased distractibility.

As for memory testing, two subtests of the first edition of the Kaufman Assessment Battery for Children (K-ABC, Dutch version) [18] were administered. The Hand Movements subtest assesses visual-motor memory. In this subtest, the child is shown different hand positions and must reproduce these. In the Number Recall subtest for verbal memory, the child must repeat a sequence of numbers of increasing length. Both tests have a mean population mean of 10 and an SD of 3. Higher scores represent better performance.

### Patient characteristics

We recorded perinatal characteristics such as the following: fetoscopic endoluminal tracheal occlusion (FETO, yes/no), inborn (yes/no; yes if born in our hospital or another CDH center), observed-to-expected lung-to-head ratio (o/e LHR) (recorded at 32-week gestational age, or measurement closest to that moment), primary closure of defect (yes/no), open repair versus minimal access surgery (MAS) (yes/no), duration of initial stay at ICU (days), cardiac malformations (yes/no; recorded if follow-up by a pediatric cardiologist was necessary), cardiac malformations for which surgical intervention is needed (yes/no), maximum vasoactive inotropic score (VIS) during first 2 months of initial stay, veno-arterial extra corporeal membrane oxygenation (ECMO) treatment (yes/no), maternal education level (MEL, low-to-middle or high, based on International Standard Classification of Education (ISCED)) [19].

### Statistical analyses

Mann–Whitney *U* tests were used to compare continuous data of participants and those of non-participants lost to follow up; chi-square tests and Fisher’s exact tests were used for categorical data. The one-sample binomial test was used to compare outcome proportions in our sample with normative proportions, and the one sample *t*-test was used to compare continuous test results with normative scores. Effect sizes were interpreted according to Cohen’s *d*. When assumptions for linear regression were met, univariable regression analyses were performed to assess associations between characteristics and neuropsychological outcome. In case of a nonparametric distribution, the Mann–Whitney *U* test was used to investigate associations between dichotomous determinants and cognitive scores and Kendall’s tau was used in case of continuous determinants. Characteristics used for association analyses were birthweight, inborn versus out-born, primary closure of defect versus patch repair, open repair versus MAS, initial ICU stay in days, maximum VIS, and MEL, as we aimed to cover perinatal characteristics, degree of illness, effects of surgical approach, and socioeconomic status. Determinants that were significantly associated with cognitive performance in univariable analyses were used in multivariable linear regression analyses. We used continuous raw scores for association analyses with NEPSY-outcome (e.g., completion time in seconds and number of mistakes) instead of percentile categories, to perform linear regression. In case variables were strongly skewed, log-transformation was considered. All statistical tests were two-sided with a significance level of 0.05. Analyses were performed by using SPSS version 25 (IBM SPSS Statistics, IBM Corporation, Armonk, NY).

## Results

### Population

**Fig. 1:**
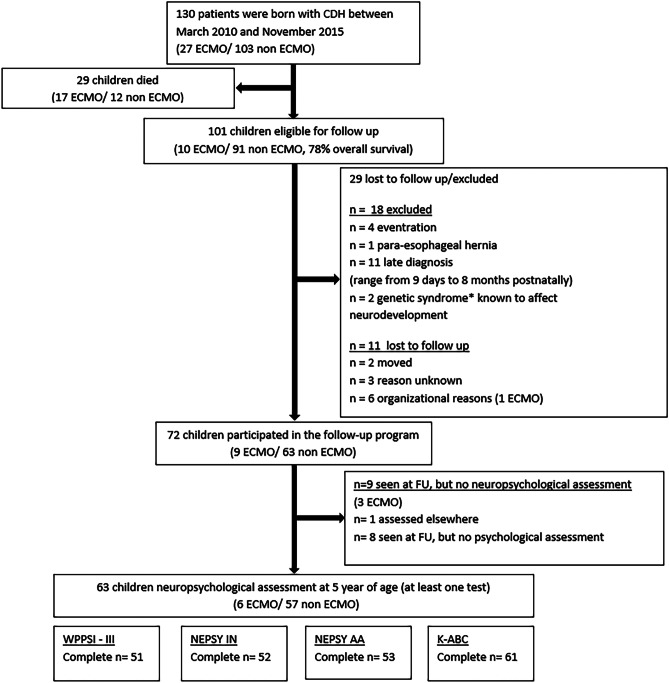
Flowchart of inclusion and exclusion of study participants. CDH, congenital diaphragmatic hernia; ECMO, extracorporeal membrane oxygenation; WPPSI, Wechsler Preschool and Primary Scale of Intelligence–III; NEPSY I-N, NEPSY Inhibition-Naming; NEPSY I-I, NEPSY Inhibition-Inhibition; K-ABC, Kaufman Assessment Battery for Children. *Chromosomal duplication, Sotos syndrome

We included 63 children who had been assessed with at least one cognitive test, either the WPPSI-III-NL, NEPSY-II-NL-AA, NEPSY-II-NL-IN, or the K-ABC visual and verbal memory (For a flowchart of inclusions, refer to Fig. 1). Fifty-one (81%) children had been assessed with the WPPSI-III-NL; fifty-two (83%) with both the NEPSY-II-NL-IN and the NEPSY-II-NL-II; fifty-three (84%) with the NEPSY-II-NL-AA; and 61 (97%) with the K-ABC. Forty-seven of the 63 children (75%) were born in a CDH center, and in 42 (89%) o/e LHR had been recorded prenatally, with a median of 47.9%. Comparison of the baseline characteristics of participants and non-participants revealed one significant difference: a higher number of children with non-Dutch ethnicity among the non-participants (Table 1).

**Table 1:** Background characteristics

	***Participants*** ***n* = *63 (76%)***		***Non-participants*** ***n* = *20 (24%)***		***p-value***
Boys	38	(60.3)	12	(60.0)	0.98
FETO	1	(1.6)	0	(0)	1.00
Birthweight, g	3000^a^	(1900–3900)	3200^b^	(2300–3775)	0.44
Gestational age, weeks	38.3^c^	(34.9–42.0)	38.3	(31.6–41.6)	0.85
Inborn	47	(74.6)	14^c^	(70)	1.00
Left-sided defect	56	(88.9)	16	(80.0)	0.45
o/e LHR %	47.9^d^	(24–89.1)	56.3^e^	(33.5–67.2)	0.30
Age at surgery, days	3	(1–14)	3	(1–15)	0.76
Primary closure of defect	23	(36.5)	6	(30)	0.56
Open repair	27	(42.9)	10^c^	(50)	0.45
Initial ventilation, days	8	(1–258)	9^c^	(1–48)	0.78
Initial ICU stay, days	16	(1–274)	19^c^	(3–59)	0.99
General anesthetic events within the first 24 months	2	(1–13)	2^ g^	(1–7)	0.57
Sepsis	13	(20.6)	6	(30)	0.40
Cardiac malformations	6	(9.5)	3	(15)	0.43
Surgical intervention needed	0	(0)	1*	(1)	1.00
Inhaled nitric oxide treatment	21	(33.3)	9^f^	(45)	0.17
Maximum VIS	10.3	(0–67.9)	12.5	(0–69.6)	0.59
Veno-arterial ECMO	6	(9.5)	3	(15)	0.68
*Time on ECMO, h*	183	(85–561)	261	(96–264)	1.00
Abnormal cranial ultrasound	7^a^	(11)	2^c^	(10)	1.00
Dutch ethnicity	50	(79)	10^c^	(50)	**0.01**
MEL					
*Low to middle*	19^ g^	(30)	3^ h^	(15)	0.62
*High*	29	(46)	3	(15)	

Data presented as median (range), or *n* (%)

Non-participants: patients lost to follow-up or seen at follow-up, but without neuropsychological assessment

*FETO *fetal endoluminal tracheal occlusion, *ECMO *extracorporeal membrane oxygenation, *ICU *intensive care unit, *MEL *maternal education level, *VIS *vasoactive inotropic score, bold: significant difference between groups. *Tetralogy of Fallot

^a^5 missing data, ^b^3 missing data, ^c^1 missing data, ^d^measured in 42 out of 47 inborn patients, ^e^measured in only 11 out of 14 inborn patients, ^f^2 missing data, ^g^15 missing data, ^h^14 missing data

### Test results

**Fig. 2:**
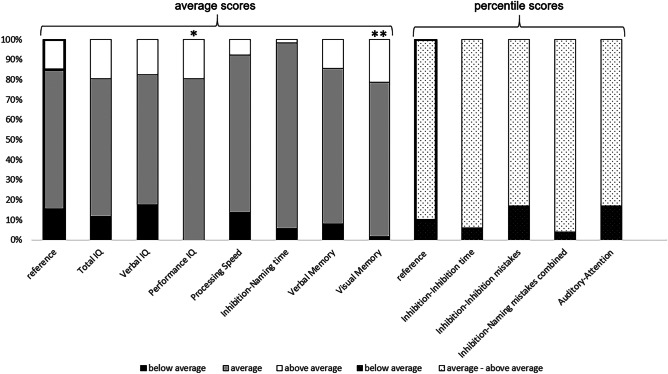
Results on neurocognitive tests in our study population compared to reference population. *p < 0.05, **p < 0.001, for an overview of scores refer to Supplemental Tables S1 and S2

Performance on all tests did not significantly deviate from the norm, except for scores on performance IQ and visual memory, which were significantly higher (Cohen’s *d* respectively 0.45 and 0.56). For details on results on all tests, refer to Fig. 2 and Supplementary Tables 1 and 2 (Table S1 Continuous scores compared to reference scores, Table S2 Percentile scores compared to reference scores).

### Univariable analysis

Longer ICU stay was associated with lower performance IQ (*B* −3.37, *p* = 0.04 (Confidence Interval (CI) −6.65 to −0.09)). Low-to-middle MEL was associated with lower verbal IQ (*B* −17.38, *p* < 0.01 (CI −26.79 to −7.98)) and total IQ (*B* −14.30, *p* < 0.01 (CI −23.41 to −5.19)) compared to high MEL.

Children who had undergone open surgery made significantly more mistakes on the inhibition-inhibition task (NEPSY-II-NL-II) than the children who had undergone minimal access surgery (MAS) (*p* < 0.001), and more mistakes were associated with lower birthweight (*τ* =  −0.25, *p* = 0.02), longer ICU stay (*τ* = 0.25, *p* = 0.01), and higher maximum VIS (*τ* = 0.28, *p* = 0.01).

The total number of mistakes during naming and inhibition (NEPSY-II-NL-IN and NEPSY-II-NL-II) was significantly higher in children treated with open surgery versus MAS (*p* < 0.001), and more mistakes were significantly associated with longer ICU stay (*τ* = 0.22, *p* = 0.03) and higher maximum VIS (*τ* = 0.27, *p* = 0.01).

For auditory attention, we found significantly lower outcome scores in relation to low-to-middle MEL versus high MEL (*p* = 0.04).

Determinants significantly associated with verbal memory were not identified, while low-to-middle MEL was associated with lower visual memory scores (*B* =  −1.52, *p* = 0.04, (CI −2.97 to −0.07)).

### Multivariable analysis

For the multivariable analysis, birthweight, ICU stay in days, maximum VIS, and open surgery versus MAS served as independent variables; inhibition and naming task scores served dependent variables.

In multivariable analysis, only open repair remained significantly associated with the number of mistakes in the inhibition task (NEPSY-II-NL-II; *B* = 5.52, *p* = 0.04, (CI 0.32–10.72)) and number of mistakes in inhibition and naming tasks combined (*B* = 7.60, *p* = 0.01, (CI 1.77–13.44)).

For further details regarding analyses, refer to supplemental files (Table S3 Univariate analyses Intelligence; Table S4 Univariate analyses Memory; Table S5 Association analyses Inhibition, Naming and Auditory Attention; Table S6 Multivariable analysis Inhibition-Inhibition, total mistakes; Table S7 Multivariate analysis Inhibition-Inhibition and Inhibition-Naming, total mistakes).

## Discussion

In this study, we tested cognitive functioning and explored possible predictors in 5-year-old CDH-survivors. Given the abundant evidence that any neurocognitive problems of CDH-survivors will eventually appear around school age [5–7, 20, 21], we hypothesized that cognitive deficits could be detected earlier in life. However, we found no significant deviations from normative scores at this age on intelligence, attention, inhibition, and verbal memory, while visual memory scores were significantly higher than normative scores. The only clinical variable that remained significant in the multivariable analysis was type of surgery; i.e., children who had undergone open repair surgery scored lower on inhibition tasks than those treated with MAS. The absence of neurodevelopmental problems in our study population may support the growing-into-deficit theory, which has it that neurocognitive problems become functionally evident with increasing age. An alternative explanation is that the tools currently available are not sensitive enough to detect these problems.

Studies on neurodevelopment in this patient population at toddler age generally reported neurodevelopment to be within normal ranges [22–24], or only slightly below [25, 26]. Most of these studies, however, used a version of the Bayley Scales of Infant Development, which is not devised for assessing specific neurocognitive functions, but rather for general development [27]. Yet, several domains of neurodevelopment around 5 years of age can be assessed with the WPPSI. Danzer and colleagues, for example, found total, verbal, and performance intelligence to be within normal ranges in 35 5-year-olds born with CDH, who, however, had significantly more low and borderline scores on at least one domain compared to normative cohorts [9]. This discrepancy with our findings might be explained by the greater number of children treated with ECMO in their group. Interestingly, the cohort studied showed favorable scores on academic achievement using assessment instruments to estimate school readiness [9]. Another study found that 44% of their CDH-patients (born 2001–2005) aged 4 to 7 scored < 80 on any of the tests of both the WPPSI and a language assessment [10]. Yet, the sample size was small (*n* = 16), and four patients had previously been diagnosed with neurodevelopmental impairment. In our study, children with intellectual disabilities or with comorbid syndromes affecting cognition were excluded.

Given the results from our and the aforementioned studies, it remains a topic of debate whether it is even possible to detect problems with higher order neurocognitive functions in children this young. As higher neurocognitive functions such as working memory gradually develop between 4 and 18 years [28], it might not be surprising that we found no obvious abnormalities at 5 years of age.

### Determinants

In univariable analysis, longer ICU-stay was negatively associated with performance IQ, which has been demonstrated before [22, 29]. Moreover, lower birthweight, higher maximum VIS, and open repair were related to poorer inhibition. In a previous study, maximum VIS was found to be related to memory performance, possibly as an indicator for severity of illness or rather as a proxy for altered brain perfusion [6]. In this study, open repair was the only clinical variable that remained significantly associated with poorer inhibition in multivariable analysis. The effect of open repair versus MAS on brain development is still a topic of debate. Yet, as open repair is mostly performed in clinically unstable patients, including the ones treated with ECMO, whereas MAS is only done in relatively stable patients with smaller defects, the association we found might reflect the extent of critical illness rather than the effect of the surgical technique on the brain. Indeed, children treated with laparotomy had a significant longer stay on the PICU (*p* < 0.01).

### Strengths and limitations

We were able to collect data from a relatively large group of children born with a rare anatomical malformation who had been prospectively included in a follow-up program. However, several limitations must be mentioned. First, o/e LHR was not included in the association analyses because up to 25% of our patients were out-born, indicating that the defect was not diagnosed prenatally, and that, therefore, o/e LHR had not been measured. The measurements that we do have show a relatively mild cohort, as nearly no children with severe lung hypoplasia are included (o/e LHR < 25%), which must be taken into account while interpreting our results. In addition, the defect size could not be reliably retrieved for every participant, as structural recording of defect size only started in recent years. Second, we excluded patients with severe genetic syndromes, but we do not know whether the others might have had small genetic defects, as exome sequencing was not yet offered in this study period [30]. Therefore, we cannot say for certain whether the possible existence of small genetic defects might have influenced our findings. Of note, recent studies have demonstrated that de novo deletions in CDH-survivors are associated with worse neurodevelopmental outcome [31]. Third, several eligible children were not assessed due to fatigue, decreased attention, or lack of motivation. Therefore, our findings might overestimate performance, as those children might have performed worse on several tasks. In addition, we lost significantly more children with a non-Dutch ethnicity to follow-up compared to the ones with a Dutch ethnicity. Fourth, although we examined a relatively large cohort of CDH-survivors, the sample might not be fully representative of the general population of CDH-survivors. In a previously studied cohort of children born with CDH (born 2006–2009), more neonates had been treated with ECMO and maximum VIS scores were higher than in our cohort [6]. The question remains whether this difference is due to a new standardized treatment protocol [32] or a natural fluctuation in disease severity [2]. As we cannot rule out that the current study cohort had a relatively mild level of disease severity, given the relatively high O/E LHR measurements and the clinical characteristics, we assume that conclusions should be interpreted with caution and that more studies in other cohorts of children with CDH are needed.

## Future directions

There is consensus that being born with CDH has an impact on neurodevelopment at school age. Yet, there is a need for predictive factors. While we merely focused on clinical characteristics, social factors such as socioeconomic background should be further explored in coming studies. Maternal education level (MEL) was relatively high in our cohort in comparison to normative data, although quite a few numbers on MEL were missing. Considering the assumed correlation of MEL and intelligence of offspring [33], we would have expected a generally high IQ among our population, instead of average. This argues for an actual effect of having survived CDH on brain development, despite the mild clinical characteristics. Another field of interest is neuroimaging markers. Recently, prenatal cerebellar diameters of CDH-patients on ultrasound were found to be smaller than the cerebellar diameters of healthy fetuses [34], which is of importance as the cerebellum is involved in many neurocognitive functions [35]. Also, given that intelligence in CDH-survivors is generally within the normal range [36], but problems occur in specific neurodevelopmental domains [6], possibly a subtle form of brain injury is reflected in those problems. Longitudinal magnetic resonance imaging (MRI) may inform us about the underlying mechanisms of growing into those deficits. For instance, previous MRI studies have shown that hippocampal volume is decreased in survivors of critical illness at school age and is associated with decreased memory function [37]. Thus, brain imaging, together with close neuromonitoring during critical moments, enables investigation of the mechanisms and trajectories of deviant brain development. This will eventually result in possible targets for intervention. Moreover, age-appropriate validated assessment instruments including up-to-date population specific reference data will always be a challenge for multicenter studies and outcome registries.

## Conclusion

We found neurodevelopmental outcome to be within normal ranges at 5 years of age within our cohort of CDH-survivors. However, the knowledge that the neurocognitive performance of these children around 8 years of age is generally not normal underscores the growing-into-deficit hypothesis in this group of children. Multiple cohort studies are imperative to account for the natural fluctuation in clinical course, and to determine whether some subgroups show signs of neurocognitive developmental problems as early as preschool age, for which timely intervention is desirable. Therefore, future research should focus on both more sensitive assessments and specific determinants of neurodevelopment in a structured follow-up program to prevent or mitigate any future neurocognitive problems in these children.

## Supplementary Information

Below is the link to the electronic supplementary material.

Supplementary file1 (DOCX 24 KB)

## Appendix

**Table:** Protocol Neuropsychological assessment CHIL 3.0 For children with Congenital Diaphragmatic Hernia, from 2023

	**Parents**	**Child/adolescent**
12 months (1 year)	*Always:* Questionnaires: Parental burden Child trauma (CATS) Parental trauma (PCL-5) *Only when necessary:* Anamnesis interview Social emotional development (questionnaire from Bayley) (40 min.)	*Only when necessary:* Developmental assessment Bayley-III Cognition and Language scales *If not possible:* ABAS-3 parental questionnaire adaptive functioning (1 hour)
30 months (2 ½ years)	*Always:* Anamnesis interview Questionnaires: Parental burden Child trauma (CATS) Parental trauma (PCL-5) Social emotional development (questionnaire from Bayley) (40 min.)	*Always:* Developmental assessment Bayley-III Cognition *If not possible:* ABAS-3 parental questionnaire adaptive functioning (1 hour)
5 years	*Always:* Anamnesis interview Questionnaires: Parental burden Child trauma (CATS) Parental trauma (PCL-5) Quality of life/Health status (DUX-25/ PedsQL) Behaviour (SDQ) Executive functioning (BRIEF-P) (60 min.)	*Always:* Neuropsychological assessment (NPA): • WPPSI-IV 4;0-6;11 years:10 subtests (TIQ and primary indexes) • NEPSY inhibition • NEPSY auditory attention (2 1/2 hours)
8 years + 12 years	*Always:* Anamnesis interview Questionnaires: Parental burden Quality of life/Health status (DUX-25/ PedsQL) Behaviour (SDQ) Executive functioning (BRIEF-2) Perceived cognitive function (Peds-PCF short) (60 min.)	*Always:* Anamnesis interview Questionnaires: Quality of life/Health status (DUX-25/ PedsQL) Perceived cognitive function (Peds-PCF short) NPA: • WISC-V-NL (8 years: 10 subtests; TIQ and primary indexes) (10 years: abbreviated version) • NEPSY Visuospatial memory • Auditory Verbal Learning Test • Beery VMI • Dot cancellation task (Bourdon) • D-KEFS Colour Word Test • D-KEFS Trailmaking Test • BADS-C 6-elements task (8 years: 4 hours; 12 years: 3 hours)
17 years	*Always:* Parental burden Perceived cognitive function (Peds-PCF short) (20 min.) *Only when necessary:* Anamnesis interview	*Always:* Anamnesis interview Questionnaires: Health status (PedsQL) Perceived cognitive function (Peds-PCF short) Behavior (Aseba Youth Self Report) (30 min.) *Only when necessary:* NPA: • WAIS-IV-NL: 10 subtests, 4 indexen • WNV Spatial Orientation • Auditory Verbal Learning Test • Dot cancellation task (Bourdon) • D-KEFS Colour Word Test • D-KEFS Trailmaking Test • BADS-C 6-elements task (3 hours)

## Abbreviations

CI: Confidence Interval
CDH: Congenital diaphragmatic hernia
ECMO: Extracorporeal membrane oxygenation
ISCED: International Standard Classification of Education
K-ABC: Kaufman Assessment Battery for Children
MAS: Minimal access surgery
MEL: Maternal education level
NEPSY: Of A Developmental NEuroPSYchological Assessment
(P)ICU: (Pediatric) intensive care unit
VIS: Vasoactive inotropic score
WPPSI: Wechsler Preschool and Primary Scale of Intelligence
