# Supplementary material for: Neuropsychological outcome in survivors of congenital diaphragmatic hernia at 5 years of age, what does it tell?
Source: Eur J Pediatr. 2022 Dec 24;182(3):1057–66. doi: 10.1007/s00431-022-04696-1 (PMC10023636; doi:10.1007/s00431-022-04696-1)
Supplement: Supplementary file 1 — Supplementary file1 (DOCX 24 KB) [file 431_2022_4696_MOESM1_ESM.docx]

SUPPLEMENTARY FILE

| **Table S1** – index scores | | | |
| --- | --- | --- | --- |
|  | mean | SD | Difference from norm |
| **WPPSI - III** | | | |
| Total IQ | 103.4 | 15.7 | p = 0.13 |
| Verbal IQ | 102.9 | 17.9 | p = 0.23 |
| Performance IQ | 105.6 | 12.6 | **p = 0.002** |
| Processing speed | 98.4 | 15.3 | p = 0.45 |
| **NEPSY - II** | | | |
| Inhibition-naming | 10.5 | 2.2 | p = 0.10 |
| **K-ABC** | | | |
| Verbal memory | 10.2 | 2.8 | p = 0.61 |
| Visual memory | 11.4 | 2.6 | **p < 0.001** |

| **Table S2** – percentile scores | | | | | | |
| --- | --- | --- | --- | --- | --- | --- |
|  | n | % | | | Difference from norm | |
| **Inhibition-inhibition, completion time** | | | | | | |
| p ≤10 | 3 | 6 | | |  | |
| p > 10 | 49 | 94 | | | p = 0.22 | |
| **Inhibition-inhibition, number of mistakes** | | | | | | |
| p ≤10 | 9 | 17 | | |  | |
| p > 10 | 43 | 83 | | | p = 0.06 | |
| **Inhibition and Naming, total number of mistakes** | | | | | | |
| p ≤10 | 2 | 4 | | |  | |
| p > 10 | 50 | 96 | | | p = 0.11 | |
| **Auditory attention, total number correct responses** | | | | | | |
| p ≤10 | 9 | | 17 |  | |  |
| p > 10 | 44 | | 83 | p = 0.07 | |  |

| **Table S3. Univariable analyses - Intelligence** | | | | |
| --- | --- | --- | --- | --- |
|  | **Verbal IQ** | **Performance IQ** | **Processing Speed** | **Total IQ** |
| **Out born** | B 0.26,  95% CI -11.85-12.38, p = 0.97 | B 4.02,  95% CI -4.34 – 12.38, p = 0.38 | B 9.50,  95% CI -0.53 – 19.53, p = 0.06 | B 2.80,  95% CI -7.82 – 13.42, p = 0.60 |
| **Birthweight, grams** | B 0.01, 95% CI -0.00 – 0.02, p = 0.16 | B 0.00,  95% CI -0.01 – 0.01, p = 0.65 | B 0.01,  95% CI -0. 00 – 0.01, p = 0.21 | B 0.01,  95% CI -0.00 – 0.02, p = 0.22 |
| **ICU stay, days*** | B -2.63, 95% CI -7.43 – 2.18, p = 0.28 | **B -3.37,  95% CI -6.65** – **-0.09, p = 0.04** | B -3.15,  95% CI -7.23 – 0.92, p = 0.13 | B -3.72,  95% CI -7.86 – 0.42, p = 0.08 |
| **Maximum VIS** | B -0.11, 95% CI -0.43 – 0.20, p = 0.47 | B -0.11,  95% CI -0.33 – 0.12, p = 0.35 | B -0.18,  95% CI -0.45 – 0.09, p = 0.18 | B -0.14,  95% CI -0.41 – 0.14, p = 0.33 |
| **Open repair** | B -5.75,  95% CI -16.24 – 4.75, p = 0.28 | B -4.89,  95% CI -12.22 – 2.45, p = 0.19 | B -5.38,  95% CI -14.36 – 3.60, p = 0.23 | B -6.10,  95% CI -15.25 – 3.10, p = 0.19 |
| **Primary closure of defect** | B -3.77,  95% CI -14.11 – 6.57, p = 0.47 | B 2.54,  95% CI -9.81 – 4.74, p = 0.49 | B 2.18,  95% CI -6.7 – 11.08, p = 0.26 | B -0.87,  95% CI -10.01 – 8.26, p = 0.85 |
| **MEL low-middle** | **B -17.38,  95% CI -26.79 – -7.98, p < 0.01** | B -7.2,  95% CI -15.33 – 0.92, p = 0.08 | B -4.75,  95% CI -14.71 – 5.21, p = 0.34 | **B -14.30,  95% CI -23.41** – **-5.19, p <0.01** |
| ** log-transformed, ICU: intensive care unit, VIS: vasoactive inotropic score, MEL: maternal education level, bold: significant association* | | | | |

| **Table S4. Univariable analyses - Memory** | | |
| --- | --- | --- |
|  | **Verbal memory** | **Visuo-spatial memory** |
| **Out born** | B -0.17,  95% CI -1.78 – 1.44, p = 0.83 | B 0.70,  95% CI -0.79 – 2.19, p = 0.35 |
| **Birthweight, grams** | B 0.00,  95% CI 0.00 – 0.00, p = 0.56 | B 0.00,  95% CI -0.00 – 0.00, p = 0.88 |
| **ICU stay, days*** | B -0.01,  95% CI -0.70 – 0.67, p = 0.97 | B -0.25,  95% CI -0.89 – 0.39, p = 0.44 |
| **Maximum VIS** | B 0.04,  95% CI -0.00 – 0.00, p = 0.13 | B 0.02,  95% CI -0.02 – 0.06, p = 0.35 |
| **Open repair** | B -0.67,  95% CI -2.10 – 0.76, p = 0.35 | B -0.67,  95% CI -2.01 – 0.67, p = 0.32 |
| **Primary closure of defect** | B -1.01,  95% CI -2.43 – 0.42, p = 0.16 | B -0.53,  95% CI -1.87 – 0.81, p = 0.43 |
| **MEL low - middle** | B -1.28,  95% CI -2.85 – 0.30, p = 0.11 | **B -1.52,  95% CI -2.97**  – **-0.07, p = 0.04** |
| **log-transformed, ICU: intensive care unit, VIS: vasoactive inotropic score, MEL: maternal education level,  bold: significant association* | | |

| **Table S5. Association analyses – Inhibition, Naming and Auditory Attention** | | | | | | | | |
| --- | --- | --- | --- | --- | --- | --- | --- | --- |
|  | **Inhibition-Inhibition  total mistakes** | | **Inhibition-Inhibition  and Inhibition-Naming  total mistakes** | | **Inhibition-Inhibition  completion time** | | **Auditory Attention  correct responses** | |
|  | Mann-Whitney U | | | | | | | |
| Open repair (yes-no) | **p < 0.001** | | **<0.001** | | p = 0.171 | | p = 0.776 | |
| Inborn (yes-no) | p = 0.07 | | p = 0.295 | | p = 0.563 | | p = 0.239 | |
| Primary closure of defect (yes-no) | p = **0.034** | | p = 0.054 | | p = 0.889 | | p = 0.816 | |
| MEL (low to middle - high) | p = 0.126 | | p = 0.055 | | p = 0.320 | | p = **0.040** | |
|  | Kendall’s Tau | | | | | | | |
| Maximum VIS | **p = 0.006** | τ = 0.28 | **p = 0.007** | τ = 0.27 | p = 0.968 | τ = 0.004 | p = 0.489 | τ = 0.07 |
| Birthweight, grams | **p = 0.016** | τ = -0.25 | p = 0.102 | τ = -0.17 | p = 0.707 | τ = -0.04 | p = 0.851 | τ = 0.02 |
| ICU stay, days* | **p = 0.011** | τ = 0.25 | **p = 0.028** | τ = 0.22 | p = 0.343 | τ = 0.09 | p = 0.610 | τ = 0.05 |
| **log-transformed, MAS: minimal access surgery, MEL: maternal education level, VIS: vasoactive inotropic score, ICU: intensive care unit, bold: significant association* | | | | | | | | |

| **Table S6. Multivariable linear regression, Inhibition – Inhibition, total mistakes** | | | | |
| --- | --- | --- | --- | --- |
|  | B | 95% CI | p |  |
| Birthweight, grams | -0.003 | ( -0.01 – 0.001) | 0.13 |  |
| ICU stay, days* | 1.28 | ( -1.62 – 4.17) | 0.38 |  |
| Maximum VIS | 0.083 | ( -0.10 – 0.26) | 0.35 |  |
| Open repair | **5.52** | **( 0.32 – 10.72)** | **0.038** |  |
| **log-transformed, VIS: vasoactive inotropic score, ICU: intensive care unit, bold: significant association* | | | | |

| **Table S7. Multivariable linear regression, Inhibition – Inhibition and Inhibition-Naming, total mistakes** | | | |
| --- | --- | --- | --- |
|  | B | 95% CI | p |
| ICU stay, days* | 1.16 | ( -2.28 – 4.59) | 0.50 |
| Maximum VIS | 0.13 | ( -0.08 – 0.34) | 0.22 |
| Open repair | **7.60** | **( 1.77 – 13.44)** | **0.01** |
| **log-transformed, VIS: vasoactive inotropic score, ICU: intensive care unit, bold: significant association* | | | |
